# Comparison between total thyroidectomy and hemithyroidectomy in TIR3B thyroid nodules management

**DOI:** 10.1007/s12020-022-03162-0

**Published:** 2022-08-20

**Authors:** Domenico Albano, Giorgio Treglia, Francesco Dondi, Raffaele Giubbini, Alessandro Galani, Carlo Cappelli, Francesco Bertagna, Claudio Casella

**Affiliations:** 1 Nuclear Medicine, ASST Spedali Civili Brescia, Brescia, Italy; 2 Department of Medical and Surgical Specialties, Radiological Sciences, and Public Health; Nuclear Medicine, University of Brescia, Brescia, Italy; 3 Clinic of Nuclear Medicine, Imaging Institute of Southern Switzerland, Ente Ospedaliero Cantonale, Bellinzona, Switzerland; 4 Department of Nuclear Medicine and Molecular Imaging, Lausanne University Hospital and University of Lausanne, Lausanne, Switzerland; 5 Faculty of Biomedical Sciences, Università della Svizzera italiana, Lugano, Switzerland; 6 Department of Clinical and Experimental Sciences, Surgical Clinic, University of Brescia, Brescia, Italy; 7 Department of Clinical and Experimental Sciences, SSd Medicina ad Indirizzo Endocrino-Metabolico, University of Brescia, Brescia, Italy; 8 Department of Molecular and Translation Medicine, Surgical Clinic, University of Brescia, Brescia, Italy

**Keywords:** Thyroidectomy, Thyroid, Thyroid cancer, TIR3B

## Abstract

**Purpose:**

Thyroid nodules classified as TIR3B according to SIAPEC 2014 are considered a clinical challenge due to the risk to be malignant. This retrospective study aimed to compare the performances of total thyroidectomy (TT) and hemithyroidectomy (HT) in the surgical management of a consecutive cohort of patients affected by TIR3B thyroid nodule in terms of side effects and the rate of malignancy detected.

**Methods:**

From 2011 to 2019, 136 (111 women, 25 men; average age of 53.5 years) patients having a thyroid nodule with a cytological diagnosis of TIR3B who underwent TT or HT were retrospectively included.

**Results:**

Out of 136 patients, 106 (78%) received TT, while the remaining 30 (22%) HT. The final diagnosis was malignant in 65 patients (48%), with follicular variant of papillary carcinoma as the most frequent. The diagnosis of malignancy was significantly more common in the TT group with 56 patients (53%) compared to the HT group with 9 cases (30%) (*p* = 0.001). Patients who underwent TT were significantly older, had larger nodules and the time between diagnosis and surgery was significantly longer compared to HT (*p* = 0.001; p0.003; *p* = 0.002). No main post-surgical complications were registered, except for one case of transient hypocalcemia in a patient who underwent TT.

**Conclusions:**

Our data showed a malignancy rate of TIR3B lesions higher than expected (48%). Both TT and HT seem to be effective approaches for the treatment of TIR3B nodules with a very low rate of post-surgical comorbidities. In the choice of surgical approach, it is crucial to consider the presence of risk factors (clinical and ultrasound characteristics), nodule size, patients’ opinion, and surgeon’s skills and experience.

## Introduction

Thyroid nodules are a common clinical challenge, due to the risk to be malignant. The risk of malignant nature is dependent on age, gender, radiation exposure history, and family history [1].

The prevalence of thyroid nodules in the general population is about 60% as documented by high-resolution ultrasonography but very few of these lesions finally demonstrate to be neoplastic (about 5%) [2] and the real challenge is to recognize this small proportion of patients with malignant nodules. There are four main tools for the assessment of thyroid nodules: clinical history and physical examination, blood measurement of serum thyroid stimulating hormone (TSH), neck ultrasonography, and, if appropriate, fine-needle aspiration (FNA). FNA allows to analyze of the cells morphology and architecture and classify thyroid nodules into categories according to the risk of malignancy [3, 4]. FNA is suggested for thyroid nodules with a diameter ≥1 cm and in presence of clinical and sonographic risk factors. Specific risk stratification systems based on ultrasound features are introduced, such as thyroid imaging reporting and data system (TI-RADS) [5]. When cytology results are indeterminate, molecular analysis of the aspirate can be suggested to avoid the need for diagnostic surgery, but these analyses are expensive and not available in all centers. Surgery is recommended for FNA findings of malignancy or indeterminate cytology when there is a high-risk condition. The choice of the kind of surgery (total thyroidectomy, TT, vs hemithyroidectomy, HT) is debated.

In 2014, the Italian Society for Anatomic Pathology and Cytology (SIAPEC) and the Italian Thyroid Association (AIT) modified the previous cytology classification, by dichotomizing the TIR3 class with two new categories, TIR3A and TIR3B, analogous respectively to the Bethesda category III (atypia of undetermined significance/follicular lesion of undetermined significance) and IV (follicular neoplasm/suspicious for follicular neoplasm) [6, 7]. The incidence of the TIR3B is <10% with a related malignancy risk rate of 20–30% [8], despite recent papers reporting higher rates up to 50–60% [9]. This increased rate may be explained by the diffusion of ultrasound examinations and the detection of less invasive forms and microcarcinoma.

This study aimed to report our experience in the surgical management of a consecutive cohort of patients affected by TIR3B thyroid nodule focusing on the comparison between TT and HT in terms of final diagnosis and post-surgical complications.

## Materials and methods

In our center, we have retrospectively evaluated 136 consecutive patients who were surgically treated from 2011 to 2019 for the presence of a thyroid nodule classified by FNAC as TIR3b according to SIAPEC 2014 classification [5]. In patients treated before SIAPEC 2014 classification publication, thyroid nodules were retrospectively reclassified. In the same period, 27 patients with a diagnosis of TIR3b were not surgically treated and followed with periodical controls due to the contraindications to surgery (*n* = 4) or refusal of the patient to perform thyroidectomy (*n* = 23).

We collected and analyzed the main epidemiological (age, gender), morphological (nodule size and location), surgical (kind of surgery and post-surgical morbidities), and histological (final diagnosis) factors, TSH before surgery, and the time interval between diagnosis and thyroidectomy.

The main indications for TT were evidence of high risk TIR3B nodule in a multinodular goiter, ultrasound suspicious nodule (considering shape, margin, echogenicity, and solid/cystic nature), nodule size > 4 cm, familiarity for thyroid carcinoma, history of radiation exposure, the presence of specific mutations suspected for carcinoma or patient’s preference for radical treatment and refusal of further eventual contralateral intervention.

Instead, the main indications for HT were solitary low-risk TIR3B nodules without previous risk factors. Patients that received HT were informed about the possibility of further surgery in case of carcinoma diagnosis.

All surgeries were performed by an experienced team in thyroid surgery.

All cytological and histological examinations after TT or HT were executed by an expert dedicated pathologist.

### Statistical analysis

The statistical analyses were performed by using MedCalc Software version 17.1 for Windows (Ostend, Belgium). Normality analysis of the data was evaluated with the Kolmogorov–Smirnov test. The numeric variables were described as average, standard deviation, and range. The categorical variables were described as simple and relative frequencies. The comparisons between patients who received TT and HT were done by using Fisher’s exact test and the chi-squared test for categorical variables and Mann–Whitney’s U-test or Student’s *t* test for continuous variables. A *p value* < 0.05 was considered statistically significant.

## Results

### Patients features

Among 136 patients, there was a prevalence of females (*n* = 111) than males (*n* = 25). Average age ± standard deviation (SD) was 53.3 ± 18 (range 18–81 years).

There was a balanced distribution of thyroid nodules with 60 nodules on the right lobe, 62 on the left lobe, and 4 in the isthmus. The mean ± SD diameter of the TIR3B nodule was 32 ± 10 mm (9–52 mm). The main characteristics of our population are described in Table 1.

**Table 1:** The main features of our population (*n* = 136)

	Average ± SD (range)	*N* (%)
Age	53.3 ± 18 (18–81)	
M:F		25:111 (18%:82%)
TSH before surgery (mU/l)	1.76 ± 0.9 (0.013–7.95)	
Nodule size (mm)	32 ± 10 (9–52)	
Nodule localization		
Right		70 (51%)
Left		62 (46%%)
Isthmus		4 (3%)
Multinodular goiter		61 (46%)
Type of surgery		
HT		30 (22%)
TT		106 (78%)
Time between diagnosis and surgery (months)	41.8 ± 12 (1–120)	
Final diagnosis		
Carcinoma		65 (48%)
Papillary		20 (31%)
Follicular variant of papillary		27 (42%)
Follicular		13 (20%)
NIFTP		4 (6%)
Poorly differentiated		1 (1%)
Benign		69 (51%)
Post-surgical morbidities		
Temporary laryngeal nerve palsy		0 (0%)
Transient hypocalcemia		1 (1%)
Hematoma		0 (0%)
Death		0 (0%)

*M* male, *F* female, *SD* standard deviation, *HT* Hemithyroidectomy, *TT* total thyroidectomy

Most patients (*n* = 106) underwent TT, while the remaining 30 patients received HT.

Among patients who received TT, 61 had multinodular goiter, 8 had a nodule with a diameter more than 4 cm, 15 had thyroid nodule with ultrasound features suspected of malignancy (for example TI-RADS 4 and 5), 1 had a previous history of radiation exposure, 2 had a familiar positive history of DTC and in the remaining 19 cases was a patient preference.

After histological examinations, carcinoma was diagnosed in 65 cases (48%): 20 classic variants of papillary carcinoma, 27 follicular variants of papillary carcinoma, 13 follicular carcinomas (11 minimally invasive and 2 widely invasive), 4 non-invasive follicular thyroid neoplasm with papillary like nuclear features (NIFTP) and 1 poorly differentiated thyroid carcinoma.

### Surgical features

The two categories (TT and HT) were well matched for gender distribution, TSH level before surgery, and nodule topography (Table 2). Age at surgery was significantly lower in patients who received HT than in patients who underwent TT (*p* = 0.011); also nodule size was significantly larger in the TT group than in HT (*p* = 0.003). In the TT group (*n* = 106), a multinodular bilateral goiter was present in 61 (57%) patients, while all patients that received HT (*n* = 30) had solitary thyroid nodules.

**Table 2:** Comparison between patients who received hemithyroidectomy and total thyroidectomy

	HT *n* = 30	TT *n* = 106	*p* value
Age mean ± SD	44.8 ± 11 (30–76)	55.7 ± 17 (18–81)	0.011
M:F	7:23 (23%:77%)	18:88 (17%:83%)	0.150
TSH before surgery (mU/l), mean ± SD	1.66 ± 0.34 (0.4–6.1)	1.79 ± 0.46 (0.013–7.95)	0.456
Nodule size (mm), mean ± SD	20 ± 6 (9–36)	30 ± 9 (10–52)	0.003
Nodule localization			0.234
Right	17 (24%)	53 (76%)	
Left	12 (19%)	50 (81%)	
Isthmus	1 (25%)	3 (75%)	
Time between diagnosis and surgery (months), mean ± SD	48 ± 23 (4–120)	24 ± 15 (1–50)	0.002
Final diagnosis Carcinoma	9 (30%)	56 (53%)	0.001
Length of hospitalization	2.6 (1–6)	2.9 (1–7)	0.126
levothyroxine replacement therapy	24/30 (80%)	106/106 (100%)	0.001
Post-surgical morbidities			
Temporary laryngeal nerve palsy	0 (0%)	0 (0%)	0.945
Transient hypocalcemia	0 (0%)	1 (1%)	0.787
Hematoma	0 (0%)	0 (0%)	0.945
Death	0 (0%)	0 (0%)	0.945

*HT* hemithyroidectomy, *TT* total thyroidectomy, *M* male, *F* female, *SD* standard deviation

The average time interval between diagnosis and surgery was significantly shorter in patients who performed TT in comparison with those who performed HT (24 vs 48 months; *p* = 0.002).

The final diagnosis of malignancy was significantly more frequent in the TT group with 56 patients (53%) compared to the HT group with 9 cases (30%) (*p* = 0.001). The nine patients of the HT group with carcinoma were subsequently treated with contralateral surgery within a mean time of 50 days (range 25–96 days).

No cases of distant metastases were discovered in all patients affected by the carcinoma.

Intraoperative neuromonitoring was used in about half of the patients (*n* = 70): 49 of the TT group and 21 of the HT group. No significant differences were available considering the length of hospitalization between the two surgical groups. Only one main post-surgical morbidity was described: a case of transient hypocalcemia in a 57 years old patient who performed TT was observed, then solve in a few weeks. No temporary laryngeal nerve palsy or hematoma were registered in both groups. No exitus was registered due to the surgery. All patients after TT received levothyroxine replacement therapy after thyroid surgery, while among HT patients 24/30.

## Discussion

TIR3B group contains those nodules with mild/focal nuclear atypia suggestive of papillary carcinoma and therefore considered to be at higher risk of malignancy, especially compared to TIR3A. The real risk of this category is difficult to estimate, with a rate of 20–30% reported in the first works [6–9], subsequently raised up to higher rates in more recent papers [10–14].

For this reason, the choice of the extent of surgery (HT or TT) in these patients is crucial and under debate.

The last American Thyroid Association (ATA) guidelines in Recommendation 19 [15] said that for patients with a solitary, cytologically indeterminate nodule, HT should be the recommended initial surgical approach, but this tool can be changed to more extent surgery if preset several epidemiological, clinical or sonographic features, and/or molecular testing when performed or patient’s preference. These risk factors include the presence of mutations specific for carcinoma, nodule size > 4 cm, ultrasound high-risk nodule, familiar history of thyroid carcinoma, or history of radiation exposure.

Unfortunately, molecular analyses are not always available and are expensive and this limits the risk analysis usually referred only to other clinical and ultrasound evidence.

The kind of initial surgical intervention should contemplate tumor size, but must also take into account all the risk factors, which are paramount in the choice of the type of treatment. HT can have some advantages, such as the theoretical low risk of surgical complications and the no need for replacement thyroid hormone therapy, and can represent a valid option. However, even in the absence of any other risk factors, it is described as an increased risk of local recurrence and worse overall survival in patients with thyroid nodule sizes between 2 and 4 cm who received HT [16].

In our analysis, most patients (78%) underwent TT; the choice of this surgical approach is supported by the increased rate of malignant thyroid lesions at final diagnosis (48%), compared to previous data available in the literature (20–30%) [6–9] and similar to a recent meta-analysis [17]. Another reason for the prevalence of TT was the patient’s preference. Some patients prefer a less extensive surgery with the realization that a second operation may be necessary if thyroid cancer is the final diagnosis. Other patients may not want to look at the possibility of a second surgical procedure and choose TT as the first step.

Although, this study was conducted in a referral center for endocrine disease, particularly thyroid diseases, and the availability of a multidisciplinary team dedicated (Thyroid Unit) with a specific attitude toward the diagnosis and the treatment of thyroid cancer. From a surgical point of view, this means a reduced risk of surgical side effects and length of hospitalization. Moreover, in our center, only a few surgeons (three) with high experience in thyroid disease surgery (more than 30 surgical interventions per year) performed thyroidectomy. From a pathologist’s point of view, this might imply a more appropriate classification in the TIR3B group only of thyroid nodules presenting real atypia in the follicular proliferation, with a consequent selection of those patients with a high suspicion of cancer and consequently exclusion of those with low-risk characteristics.

Some points in favor of TT are the treatment of potential multicentric tumors, improved disease-free survival, reduced risk of local recurrence, improved potential detection and elimination by radioiodine treatment of persistent/metastatic disease if indicated, let thyroglobulin measurement as effective, reduced risk of a second surgery.

In our study, there was a significant difference in patients’ age between the two groups. This difference may be explained because the younger patients chose to undergo the HT to have less risk of the necessity of thyroid hormone replacement therapy, considering the longest life expectancy.

Also, the time interval between diagnosis and surgery was significantly different between the two groups and was quite long mainly in HT (48 months). This long interval is due to the long waiting list of our center, where beside thyroid disease also other diseases more aggressive and dangerous are treated by surgeons. Moreover, sometimes it is the patient’s desire to wait and see and post-pone the surgical event.

All patients that received HT and had a subsequent diagnosis of carcinoma performed a contralateral surgery as completion, despite recent guidelines had a more conservative approach recommending reducing overtreatment [15]. However, most of these patients (*n* = 6) were treated before the year of publication of ATA guidelines, and the remaining three patients shared with the surgeon the intervention.

Regarding the post-surgical complications, the risks related to thyroid surgery clearly depend on several factors, such as the extent of surgery, whether this is the first or second operation, the features of the thyroid disease present, the presence of co-existing comorbidities, and the skills and experience of the surgeon [16, 18]. It should be shareable to perform thyroidectomy surgery in a center and a surgeon with high experience in this disease [19].

In a recent article, Chen et al. demonstrated a similar quality of life between TT and lobectomy in thyroid cancer [20].

In our analysis, no significant difference in surgical complications between TT and HT groups was present, and only one case of transient hypocalcemia was described. Moreover, in the 9 patients that received HT as the first approach and subsequently a second surgical operation, no complications were discovered.

Moreover, also considering the length of hospitalization the two groups are quite similar.

The decision on what is the best surgical treatment to propose to TIR3B patients, although affected by different variables and categories of risk, is both for the surgeon and patient a debated issue [20]. Shared decision-making is fundamental in this situation, taking into right account patients’ own preferences and giving them all the knowledge for the choice.

Our study presents some limitations: first) the retrospective design of the study; second) the relatively low number of patients included, also due to the rarity of the disease studies; third) the potential heterogeneous management of the patients due to the different surgical and clinical approaches during the period of inclusion.

In conclusion, our data showed an increased malignancy rate of TIR3B lesions (48%) than expected. This evidence could influence the surgical choice suggesting a radical approach by TT differently from what is stated by ATA guidelines.

The surgical management of patients with TIR3B thyroid nodules remains controversial. Both HT and TT seem to be adequate approaches with no significant post-surgical complications.
